# Law and Malpractice

**Published:** 1883-01

**Authors:** 


### LAW AND MALPRACTICE.

A recent suit for slander in one of our highest courts illustrates some points of interest to the medical profession, and also to all citizens who desire law and justice to be synonymous terms.

The facts in the case are briefly these: A prominent surgeon of this city, of large and mature experience in both civil and military surgery, is called by a practitioner to a neighboring town to the case of a young man, injured by the cars. Shortly after the surgeon's arrival, and twenty hours after the time of injury, the right arm of the patient is amputated near the shoulder. A few days after the operation, a so-called eclectic physician, a graduate of the Payne school of Philadelphia, accompanied by one or more members of the patient's family, visits this city, bringing the amputated arm, and solicits the opinion of several of our city surgeons in regard to the necessity for the operation. As a result, the eclectic is sued for slander by the surgeon who performed the operation, and in turn the surgeon is sued by the relatives of the young man for malpractice.

In the suit for slander, the trial of which occupied a week or more, the plaintiff sought to establish slander, to which the defendant replied by an allegation of malpractice. The plaintiff replied by asserting the necessity for amputation. It is only the second and third parts of these proceedings, viz., the medical, to which we desire to direct attention.

The history of the case, as elicited by the trial, is as follows: A young man, under twenty-one years of age, while coupling cars, had his arm caught between the buffers. A physician is immediately summoned, who accompanies the injured man to his home, and, upon reaching the house, another physician is called in consultation. Appreciating the serious nature of the injury, the city surgeon is called. Before resorting to surgical measures, another physician is summoned. These four medical men testify unanimously as to the necessity for the amputation. The eclectic physician who was allowed to examine the case is the only one of the five physicians who failed to see the imminent necessity for amputation.

After the lapse of about two weeks, the amputated arm is examined by several surgeons. The bones of the arm and forearm are produced in court. They exhibit numerous injuries in the region of the elbow joint, pieces of bone being split off in several places. The question turns upon the condition of the circulation in the injured arm at the time the operation was determined upon, the defense claiming that the arm was simply caught so as to pinch the back of the arm in the middle of the triceps muscle, and that the arm, for this reason, was not seriously injured and should have been saved. The plaintiff argues, the condition of the bones proved that the arm was injured at the elbow; the circulation had ceased for a number of hours; incipient mortification was present, and therefore the life of the patient depended upon immediate amputation. The testimony of four physicians, and the attendants who were present after the patient reached his house, was to the effect that the arm was distended to double its normal size by extravasated and infiltrated blood. The testimony of the surgeons to whom the arm was subsequently submitted for examination was very contradictory as to its condition and the extent of the injury. Some testify positively that the bones were not injured, and one of the number swore that he failed to find any bony lesion; another, that the arm showed that it had not been swollen, and that three weeks after the operation he could judge as to the necessity of amputation of the injured member; others testified as to the destruction and absence of important veins and nerves, and to serious injuries to bones, and also maintained that the medical men, examining the arm prior to the amputation, are, *par excellence*, the proper judges of the case.

From this mass of conflicting medical testimony a jury of apparently far less than average intelligence, and certainly with no technical knowledge, is called upon to decide between the true and the false, the law and justice; indeed, to weigh the facts in the case and render a just verdict. Was there ever a more stupendous farce? The questions of some of the jurors, without considering the verdict they rendered, stamp the trial as a comedy of errors, with ignorance and prejudice for the prominent factors. Is this a trial by one's peers, or is it a travesty on justice?

